# When I say … liberation pedagogy

**DOI:** 10.1111/medu.70223

**Published:** 2026-04-05

**Authors:** Ugo Caramori, Marco Antonio de Carvalho‐Filho

**Affiliations:** ^1^ School of Medical Sciences University of Campinas (UNICAMP) Brazil; ^2^ Research Program LEARN (Lifelong Learning, Education, Assessment and Research Network) University Medical Center Groningen, University of Groningen the Netherlands

## Abstract

Liberation pedagogy by Paulo Freire, a Brazilian educationalist, shows how critical pedagogy can illuminate identity, power, and freedom in contemporary #MedEd

Liberation pedagogy is an educational approach that seeks to expand learners' capacities to act autonomously in the world, confront structural constraints and pursue social justice. It draws on critical and emancipatory pedagogies, particularly the work of Brazilian educator Paulo Freire,^1^ who understood education as a process of fostering critical consciousness. While critical pedagogy focuses on revealing the structures of domination, liberation pedagogy emphasizes the transformative process that should follow critique. In medical education, this perspective is relevant for navigating complex power structures and adaptive systems.^2^

‘Understanding the world comes before interpreting the word’. ‐ Paulo Freire

When we say ‘liberation’, we refer to two related meanings. In Portuguese, ‘*libertação*’ (liberation) is not only release from constraint; it also signals movement toward “*liberdade*” (freedom), which Freire described as an ethical and lived goal.^3^ Freedom is often defined as the absence of interference. However, liberation presupposes productive struggle. It involves confronting the forces that shape and restrict one's reality. Liberation pedagogy acknowledges that freedom is historically and structurally constrained, while seeking to expand what becomes possible within those constraints. Liberation cannot be reduced to critique. Critique exposes structures of oppression; liberation requires action to transform them.

Engaging in liberation requires educators to navigate tensions. Their role is not to provide immediate answers but to facilitate dialogues that question assumptions and expand understanding.^1^ Freire's assertion that we must ‘read the world before the word’ underscores this stance. No curriculum can address suffering, poverty or exclusion without engaging with the social realities that produce these experiences of injustice.

In medical education, reading the world occurs when teachers guide learners to situate their clinical reasoning beyond biomedical facts and examine the structural influences that shape illness. For example, caring for a person with uncontrolled diabetes requires addressing food insecurity, housing instability (including having a fridge to store insulin) and barriers to accessing healthcare. The educational aim extends beyond identifying social determinants to recognising that clinical practice occurs within these social conditions and that as a citizen who is also a healthcare provider, fighting to change these social conditions for the better is part of becoming a doctor. Learners are not neutral observers of structural inequity; they act within systems that can either reproduce or challenge that inequity. Liberation pedagogy, therefore, invites reflection on how professional decisions, institutional norms and clinical routines shape (and often constrain) health outcomes. The teacher's role is to sustain and nurture this inquiry rather than resolve it on behalf of learners.

Liberation pedagogy also influences how role modelling is enacted in clinical supervision by allowing curiosity to guide the learning process. If a student pauses at a patient's silence, sensing that something unspoken resists translation into medical language, teachers committed to liberation do not immediately intervene; instead, they ask curiously, ‘What do you see here?’ Such moments challenge the clear‐cut grammar of certainty of some educational practices. They emphasize that knowledge is co‐created, that truth emerges through dialogue rather than being simply delivered and that meanings continue to develop in unexpected ways. They also suggest that reality is not fixed; while reading the world, we may change it.^2^

Enacting freedom in liberation pedagogy requires challenging the normalization of hierarchy, internalization of illegitimate authority and unexamined conventions that shape professional judgement. Freire described this process of alienation as a culture of silence.^3^ In medicine, silence may be reinforced by its colonial legacies, neoliberal framings of productivity and forms of epistemic injustice.^4^

Addressing this culture requires more than identifying those who are silenced. Pedagogy becomes political not because it debates politics but because it makes speaking possible.^5^ Breaking this silence requires both listening differently and questioning assumptions that present hierarchy as natural or inevitable. For example, creating structured spaces where minoritised and stigmatised learners can express how institutional practices impact them while ensuring that their voices inform curricular, evaluative and organizational changes aimed at enhancing belonging and equity. Similarly, in the context of a medical curriculum that reproduces epistemic injustice, changing this culture of silence calls for co‐developing core content with marginalised and vulnerable communities to ensure that their knowledge systems are not only legitimised but also valued by students and teachers. In liberation pedagogy, critique becomes an action to promote institutional transformation.

And yet, liberation is never final. It is not a destination but an ongoing praxis. As Freire warned, reflection without action becomes pure verbalism, and action without reflection becomes purposeless activism.^3^ The practice of freedom requires a continuous cycle of reflection and action. Critical awareness informs what we do, and action reshapes how we understand the world. Education is never neutral: Studying anatomy involves confronting the history of whose bodies were examined, and discussing health systems involves acknowledging whose lives those systems exclude. Liberation pedagogy is not about promoting guilt but cultivating responsibility.

In this paradigm, teaching involves accepting the risk of incompletion. It is not about controlling outcomes but about expanding horizons. This hopeful attitude avoids both cynicism and naïve optimism. It recognizes power and challenges that power in the face of injustice, inviting democratic dialogue and collective action to reshape institutional practices. This attitude recognises that teaching and learning happen within institutions and clinical spaces where students and patients renegotiate their voices and agency.^2^ Freedom, in this sense, does not entail standing apart from the world's wounds and injustices. Freedom is the capacity to dwell within them without surrendering, to act within them, with others, toward realising more justice. Liberation is, therefore, to teach as if the world could still be otherwise and to study as if learning were itself an act of social repair and reconciliation.

When we say liberation pedagogy, we are not invoking revolution or a fixed doctrine. We are naming a practice of hope, a discipline of listening and a politics of becoming.^3^ Naming the quiet, radical work of those who, in classrooms and clinics, refuse to treat education as a rehearsal for obedience. To those who read the world aloud and, in doing so, invite us to write it anew.

## AUTHOR CONTRIBUTIONS

**Ugo Caramori:** Conceptualization; writing—original draft; validation; writing—review and editing. **Marco Antonio de Carvalho‐Filho:** Writing—original draft; conceptualization; validation; writing – review and editing.

## Data Availability

Data sharing not applicable to this article as no datasets were generated or analysed during the current study.
